# Supplementary material for: Cryo-EM structure of the folded-back state of human β-cardiac myosin
Source: Nat Commun. 2023 May 31;14:3166. doi: 10.1038/s41467-023-38698-w (PMC10232470; doi:10.1038/s41467-023-38698-w)
Supplement: Supplementary file 1 — Supplementary information [file 41467_2023_38698_MOESM1_ESM.pdf]

Supplementary Information

Cryo-EM structure of the folded-back state of human β-cardiac myosin

Alessandro Grinzato[1,#], Daniel Auguin[2,3,#], Carlos Kikuti[2], Neha Nandwani[4,], Dihia Moussaoui[5], Divya Pathak[4], Eaazhisai Kandiah[1], Kathleen M. Ruppel[4,6,&], James A. Spudich[4], Anne Houdusse[2,&], Julien Robert-Paganin[2,&]

[1] CM01 beamline. European Synchrotron Radiation Facility (ESRF), Grenoble, France.

[2] Structural Motility, Institut Curie, Paris Université Sciences et Lettres, Sorbonne Université, CNRS UMR144, F-75005 Paris, France

[3] Laboratoire de Biologie des Ligneux et des Grandes Cultures, Université d'Orléans, UPRES EA 1207, INRA-USC1328, F-45067 Orléans, France

[4] Department of Biochemistry, Stanford University School of Medicine, Stanford, California 94305, United States

[5] BM29 BIOSAXS beamline, European Synchrotron Radiation Facility (ESRF), Grenoble, France.

[6] Department of Pediatrics, Stanford University School of Medicine, Stanford, California 94305, United States

[#] Contributed equally.

[&] Correspondence: julien.robert-paganin@curie.fr (J.R.P.), kmer@stanford.edu (K.M.R), anne.houdusse@curie.fr (A.H.).

Supplementary Figures

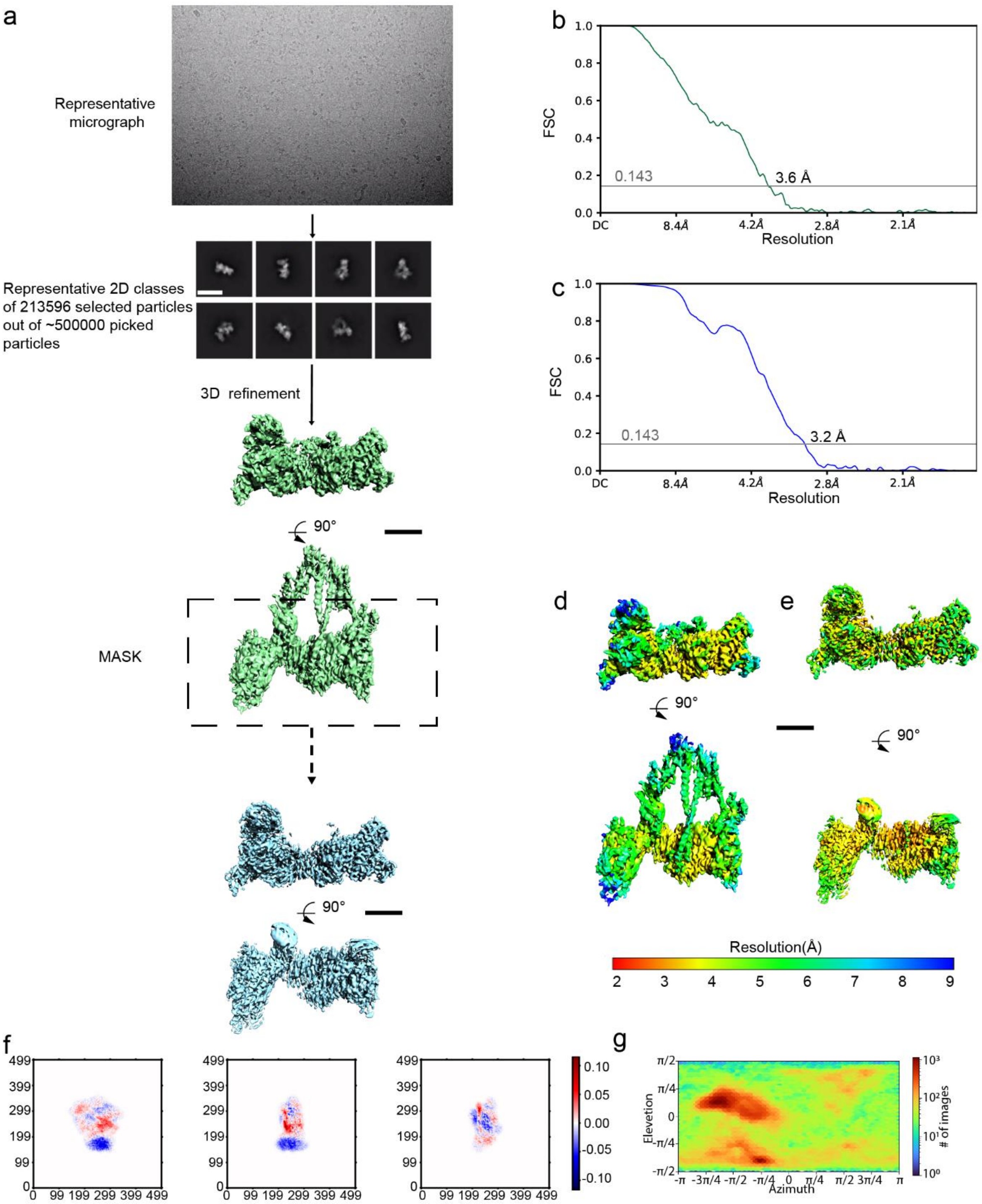

<u>Supplementary Figure 1</u> *<u>– Cryo-EM map and validation.</u>* ***(a)*** *Shows the steps of processing. A representative micrograph on gold grids, 2D classes, and the steps of refinement and masking.* ***(b)*** *and (c) show the Fourier Shell Correlation and the overall resolution on Map 1 and 2 respectively (criterion 0.143).* ***(d)*** *and* ***(e)*** *display the local resolution for each map with two orientations.* ***(f)*** *plots the angular particle distribution. Each square represents (from the left to the right) the central slice along the zy, xz and xy planes respectively. The values are shown in voxel.* ***(g)*** *Shows the flexibility of the density map shown in d.*

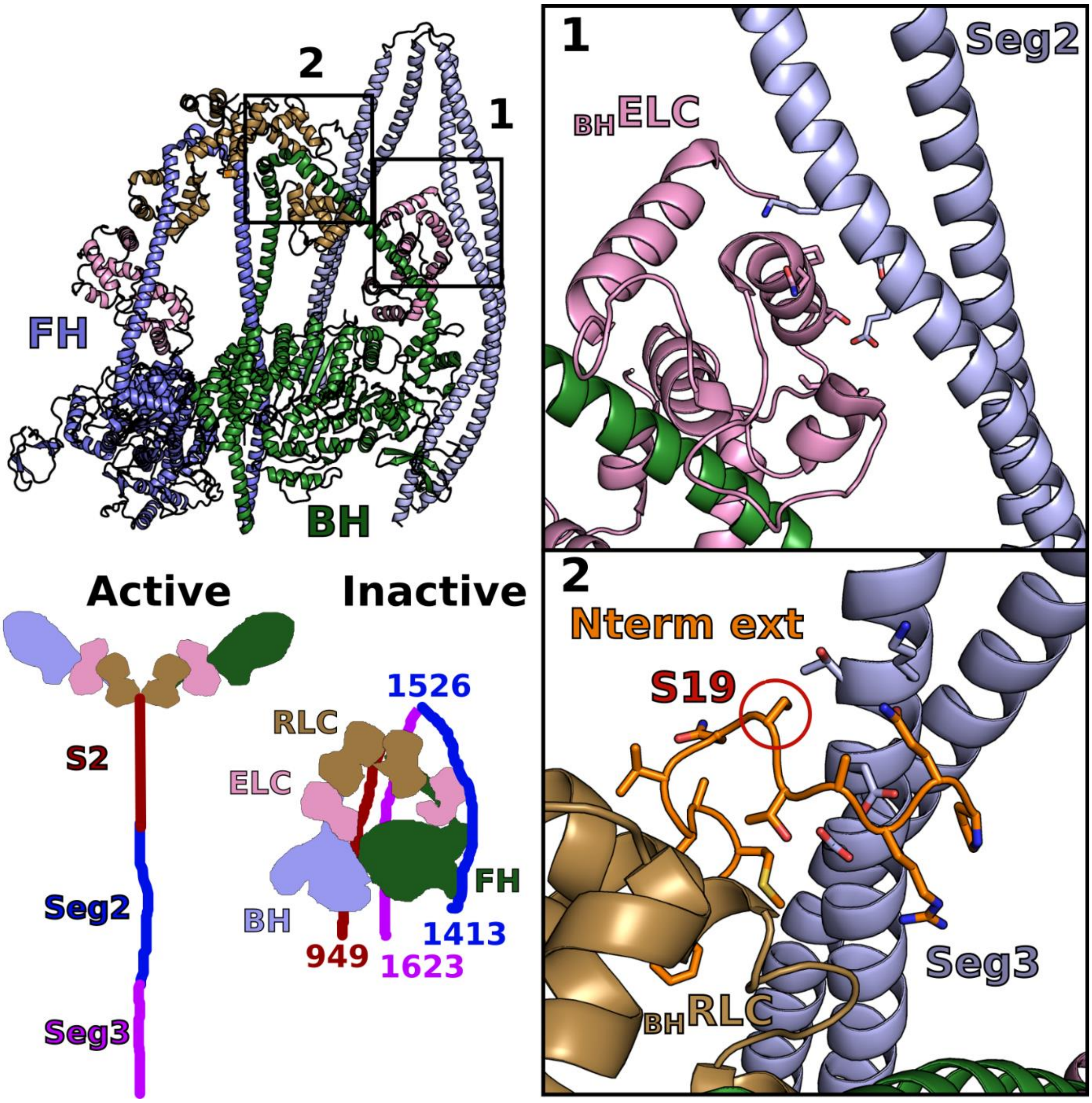

<u>Supplementary Figure 2</u> *– <u>Segments 2 (Seg2) and 3 (Seg3) coiled-coils provide additional stability in the Smooth muscle myosin 2 (SmMyo2) IHM structure</u>. (Left upper) The overall structure is presented with boxes showing the regions enlarged in the right panels. (Left lower) A cartoon shows the representation of the full auto-inhibited smooth muscle myosin, with S2, Seg2 and Seg3 coiled-coils represented as lines. An unfolded SmMyo2 is also represented. (Right upper) The Seg2 coiled-coil interacts with the ELC from the blocked head (BH) (interface 1). (Right lower) Seg3 interacts with the N-terminal extension (N-term ext) from the RLC from the BH (interface 2). The phosphorylatable serine (S19) in the $_{BH}$RLC is part of the interface with the Seg3. IHM model used here: PDB code 7MF3[1]. In the 7MF3 structure,: S2 corresponds to residues 856-949; segment 2 (Seg2) residues ~1413-1526; and segment 3 (Seg3) corresponds to residues ~1527-1623.*

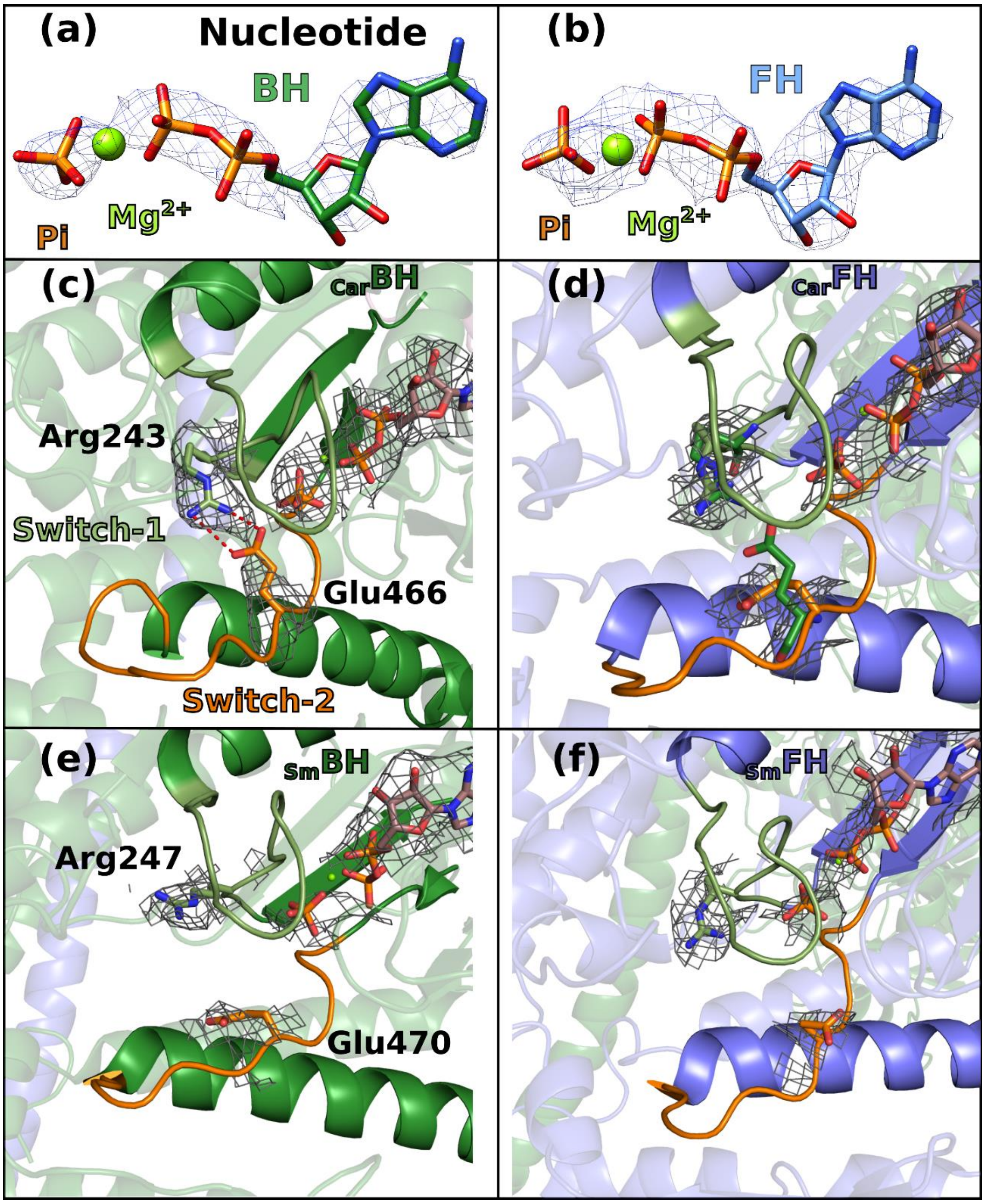

Supplementary Figure 3** – Cryo-EM density map of the nucleotide.* *Nucleotide fitted in their density map with respectively a cross-correlation of 0.7657 in the blocked head (BH, chain A)* ***(a) *and 0.890 in the free head (FH, chain B)* ***(b)***. ***(c)*** *and* ***(d)*** *represent the P_i "backdoor" in cardiac BH ($_{Car}$BH) and cardiac FH ($_{Car}$FH). The backdoor is located between Switches-1 and -2, consisting in an electrostatic bond between a glutamate (Glu466) and an arginine (Arg243). The cryo-EM density map is represented as a dark grey mesh (contour 11.95σ). In $_{Car}$BH, the backdoor is closed without ambiguity, in $_{Car}$FH, the density of Glu466 is not clear and would be compatible with a closed backdoor (the closed backdoor of $_{Car}$BH is represented as green residues in (c) to compare the conformation).* ***(e)*** *and* ***(f)*** *represent the backdoor (Arg247 and Glu470) in smooth muscle myosin BH ($_{Sm}$BH) and $_{Sm}$FH respectively. The backdoor is opened without ambiguity in $_{Sm}$BH and $_{Sm}$FH. The cryo-EM density map is represented at a contour of 13.30σ. The heads are superimposed on the motor domain (1-710 in Car, 1-722 in Sm).*

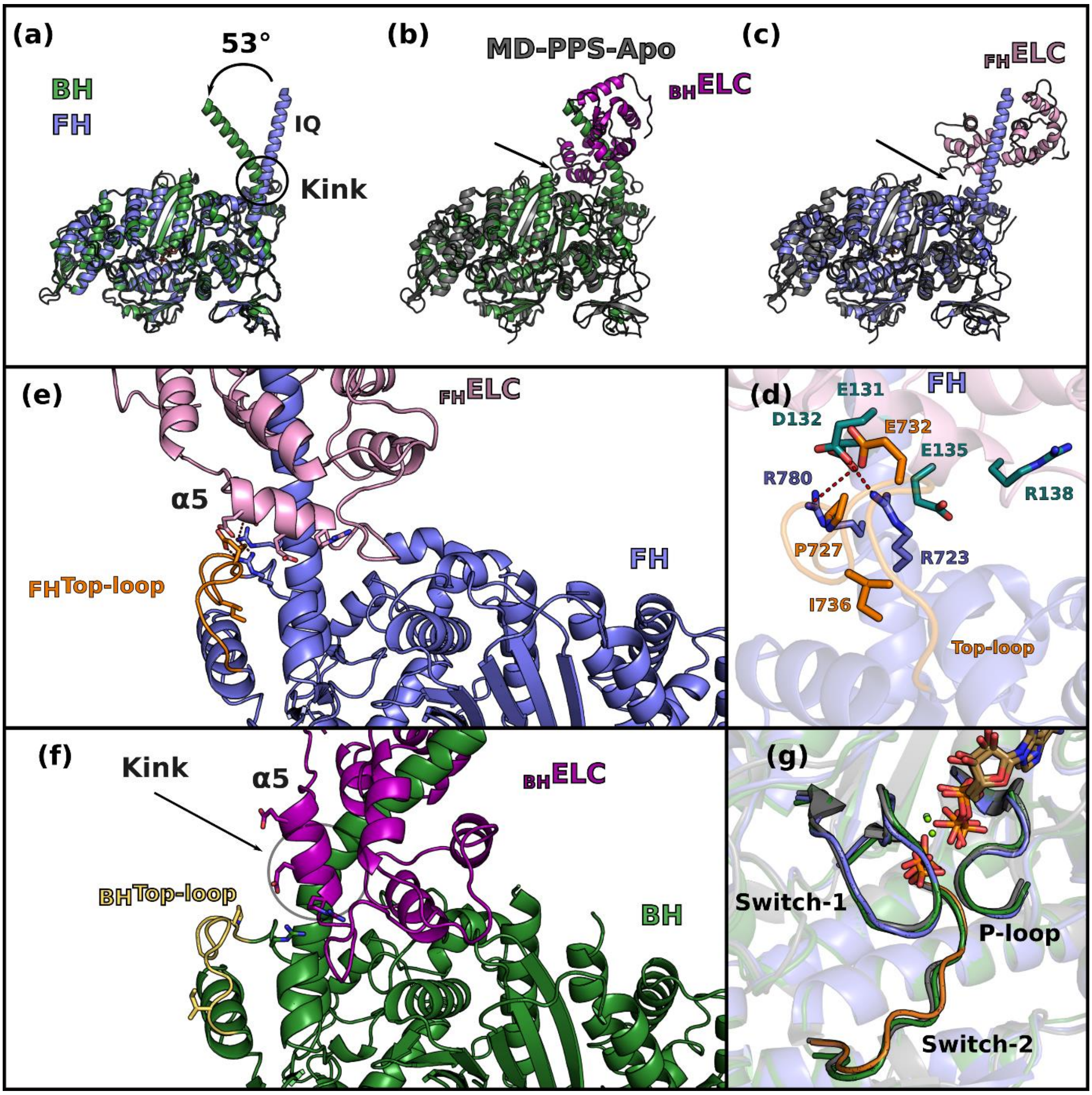

Supplementary Figure 4** – Comparison of the sequestered state with the pre-powerstroke (PPS) state of cardiac myosin.* ***(a) *The blocked head (BH, in green) and the free head (FH, colored in blue) superimpose on the motor domain with a RMSD of 0.7 Å, the only difference being the kink at the Pliant region in the lever arm, resulting in an angle of 53° between the IQ1 helix of the BH and the FH heads.* ***(b)*** *and* ***(c)*** *show the superimposition of the BH and of the FH respectively with the apo cardiac MD structure in the PPS state (colored in black, MD-PPS-Apo, PDB code 5N6A,[2]). Both motor domains superimpose with MD-PPS-Apo with a rmsd of 0.84 Å. The arrows show the ELC/Converter interface which is altered by the kink in the BH.* ***(d)*** *In the FH, the Converter/ELC interface is maintained by "musical chairs", a set of labile charged residues located on the ELC (deep teal cyan) or in the Converter, more specifically in the Top-loop[3] (orange, aa 724-738).* ***(e)*** *and* ***(f)*** *compare the motor/ELC interface and how it is completely altered by the kink present in the BH, the side chains of the musical chairs are represented as sticks on both panels. The kink of the BH shifts the position of the helix. In the BH, the interface is altered by the kink, generating a new interface between the motor (green with Top-Loop in light orange) and the ELC (purple).* ***(g)*** *BH, FH and PPS-S1-OM have all a closed active site in the inner cleft region.*

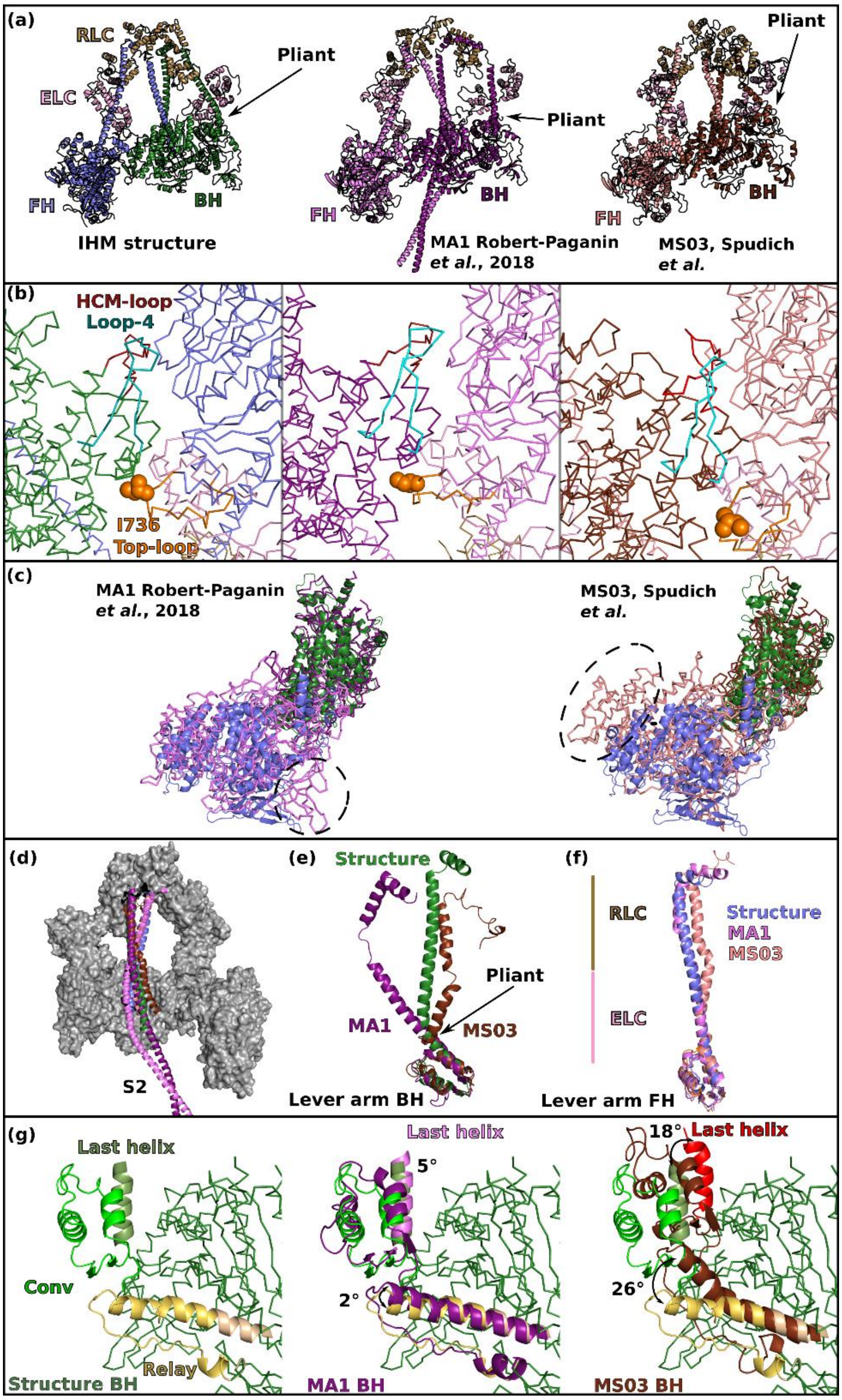

<u>Supplementary Figure 5</u> – <u>*Comparison of the IHM structure with the previous homology pseudo-atomic models.*</u> ***(a)*** *Overall representation of the IHM structure of cardiac myosin with two models, MA1*[3] *and MS03*[4]*. The pliant region of the BH is shown with an arrow.* ***(b)*** *Zoom in the head-head interface region showing that the contacts between heads were not correctly modeled.* ***(c)*** *The relative orientation of the two heads varies in the structure and in the models. Heads dimers are superimposed on the motor domain of the BH (residues 3-624) and the view is chosen to evaluate how the FH differ in position. Boxes indicate the regions chosen to zoom on the main differences in the position of the FH heads.* ***(d)*** *S2 coiled-coil in the structure and in the models interacts with the BH.* ***(e)*** *and* ***(f)*** *Comparison of the lever arm conformations of the BH and the FH respectively in the structure and in the models. The regions where the ELC and the RLC bind are indicated.* ***(g)*** *Comparison of the orientation of the Relay and the Converter in the BH. The structure (left) is superimposed with the two models (middle MA1 with Converter purple; right MS03 with Converter brown and last helix in red) for comparison. Note the pre-prestroke position of the Relay (brown) and the Converter (brown, red) in the MS03 model. In* ***(a)****, the structures are superimposed on the entire model, in (****c****), the structures are superimposed on both heads (which include both Motor domains and the lever arms including the two LCs), in* ***(e)*** *and* ***(f)*** *the lever arms are superimposed on the Converter (residues 708-777), in* ***(g)****, the structures are superimposed on the N-term subdomain (residues 3-202).*

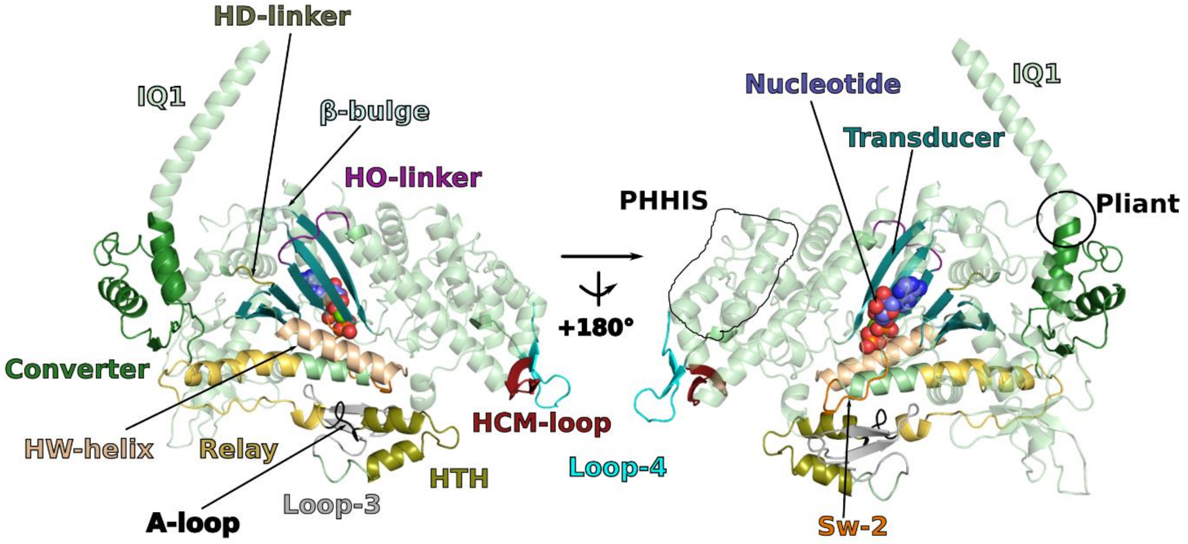

<u>Supplementary Figure 6</u> *<u>– Elements and connectors of β-cardiac myosin involved in the interfaces stabilizing the IHM.</u> The different elements are represented on the blocked head of cardiac IHM. The nucleotide indicates the position of the active site while elements of the actin interface are represented: HTH: helix-turn-helix, A-loop: activation-loop, HCM-loop, Loop-4 and Loop-3. Also represented are Switch-2 (Sw-2), IQ1 helix: HC sequence involved in binding the ELC after the pliant region. The Transducer (blue) corresponds to the central β-sheet as well as structural elements linked to this β-sheet (β-bulge, HO-linker, HD-linker). The PHHIS is part of the U50 subdomain while the HTH and Loop-3 are part of the L50 subdomains. The color coding of each part is defined on the left panel where the name of each subdomain/region is colored differently.*

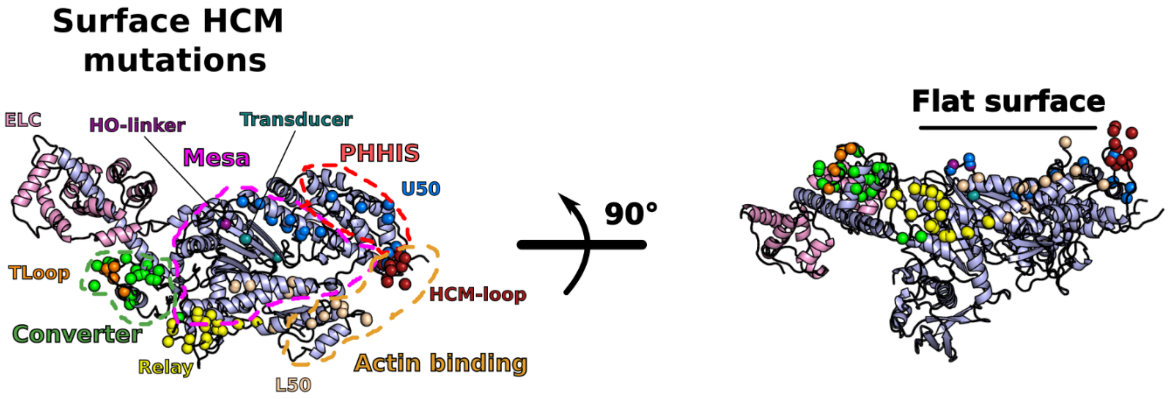

Supplementary Figure 7 *– Definition of the myosin "Mesa" and elements involved in the interfaces of the IHM, as defined by*[5]*. Surface HCM mutations are shown as balls and colored depending on the subdomain where they are located. The color coding of each part is defined on the left panel where the name of each subdomain/region is colored differently. The Mesa (in purple dotted lines) was defined as a large and flat surface containing conserved residues and HCM mutations (shown as spheres). The Primary Head-Head Interaction Site (PHHIS) and the actin-binding surface are shown with red and orange dotted lines respectively.*

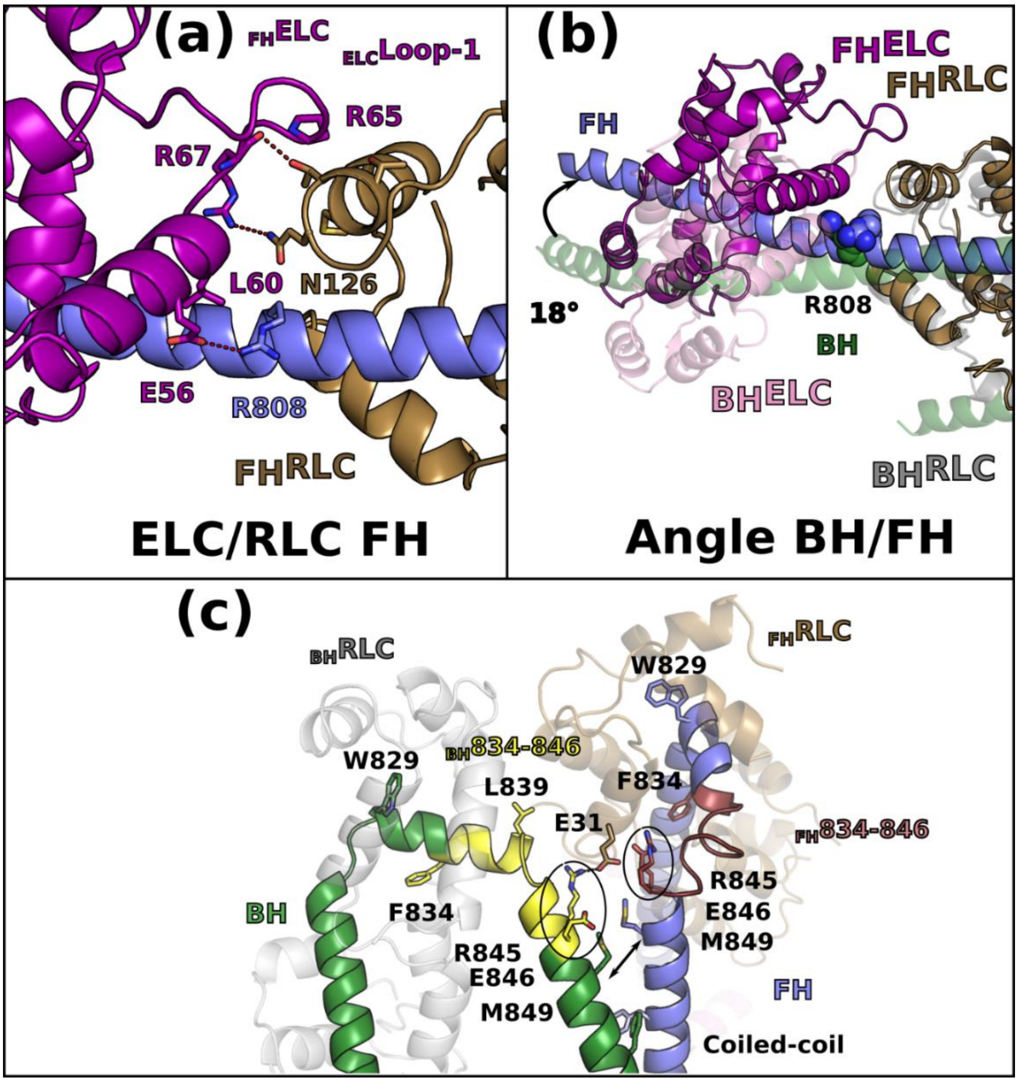

<u>Supplementary Figure 8</u> *<u>– Asymmetry in the light chain binding regions.</u> Color coding of the different elements are indicated with labels on the figures (BH: forest green, FH: dark blue, $_{FH}$ELC: magenta; $_{BH}$ELC: pink, $_{BH}$RLC: sand yellow; $_{BH}$RLC: light grey).* ***(a)*** *Interactions at the ELC/RLC interface in FH. differ from the ELC/RLC interface in BH (**Fig. 3d**). A few side chains involved in polar contacts are shown.* ***(b)*** *Lever arm of the BH and of the FH aligned on IQ2 reveals a 18° difference in the orientation as well as major differences in the ELC/RLC interface**.** **(c)** Asymmetry in the conformation and contacts of the two RLCs in the IHM structure. The RLC/IQ2 of the BH and FH heads see a different environment. This is specifically true for the region 834-848 of the heavy chain (HC), due to the fact that the coiled-coil triggers a shift in position of the HC residues (arrow between the M849 position). The HC residues (834-848) thus adopt drastically different conformations (yellow for BH, ruby red for FH) that are critical to form the RLC/RLC interface. This shift explains why some residues have different environments in the FH and in the BH: two residues characteristic of this phenomenon are contoured in black, R845 and E846. After residue R846, the phase of the coiled-coil is more canonical. Interestingly, the $_{BH}$R845 residue is involved in electrostatic interactions with the $_{FH\text{-}RLC}$E31 residue, while the $_{FH}$R845 residue is buried in the $_{FH\text{-}RLC}$N-lobe core. See also **Supplementary Movie 3** to visualize the asymmetry of this region in detail.*

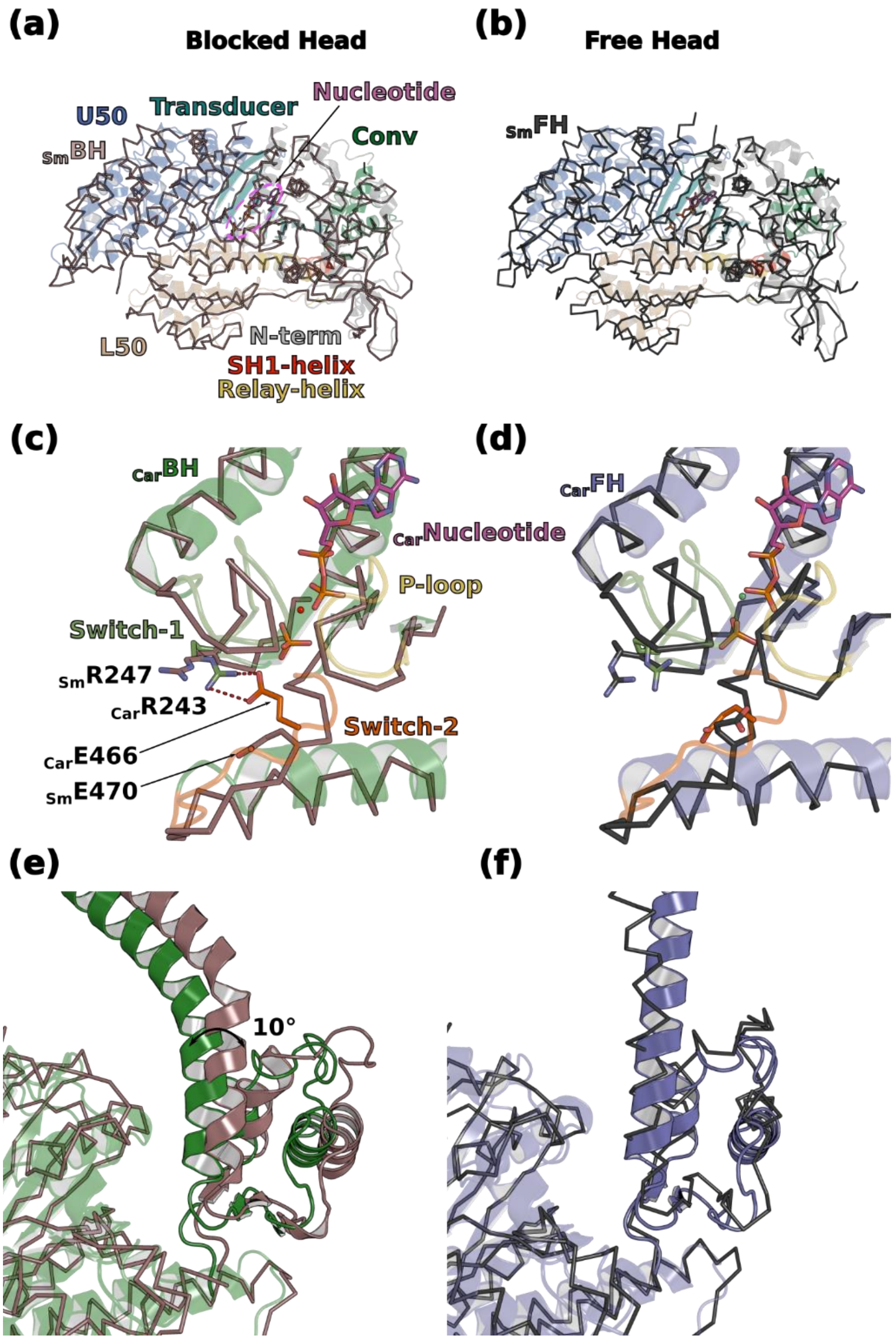

Supplementary Figure 9** – Cardiac and smooth muscle interacting-heads motifs (IHMs) differ in conformation.* ***(a) *and* ***(b)*** *shows the difference in the conformation of the motor domain of cardiac IHM ($_{Car}$IHM) (colored by subdomain) and Smooth muscle IHM ($_{sm}$IHM) (light brown for the blocked head; black for the free head).* ***(c)*** *and* ***(d)*** *show the conformation of the backdoor closing the phosphate escape tunnel[6], with $_{Car}$IHM and $_{Sm}$IHM structures depicted as in (a) and (b).* ***(e)*** *and* ***(f)*** *compare the priming of the Converter in the BH (e) and the FH (f) in $_{Car}$IHM and $_{Sm}$IHM. In* ***(a)***, ***(b)***, ***(c)*** *and* ***(d)*** *the proteins are superimposed using the N-terminal subdomain (residues 3-202); in* ***(e)*** *and* ***(f)*** *the proteins are superimposed on residues 3-707.*

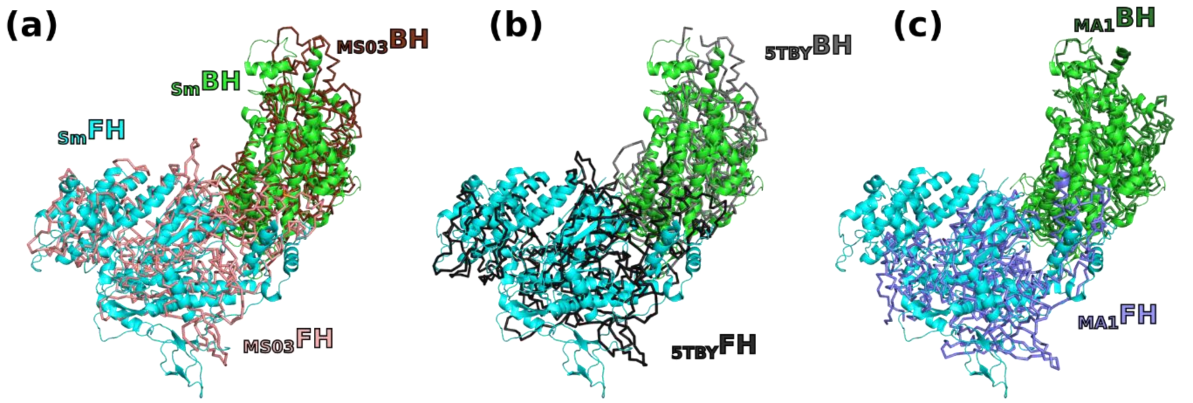

(d) **Sequence conservations**

```
Car_ELC  MAPKKPEPKKDDAKAAPKAAPAPAPPPEPERPKEVEFDASKIKIEFTPEQIEEFKEAFM 59
Sm_ELC   ------------------------------------------MCDFSEEQTAEFKEAFQ 17
Tar_ELC  ------------------------------------------MADLKAAEVEKAREHFE 17
```

$_{ELC}$Nterm N-lobe

<u>***Supplementary Figure 10*** *– the homology models MS03 and 5TBY are similar to smooth muscle myosin interacting-heads motif (*$_{Sm}$*IHM).*</u> ***(a)***, ***(b)*** *and* ***(c)*** *compare the relative orientation of the heads (free head, FH and blocked head, BH) of SmIHM to the models MS03*[4]*; 5TBY*[7] *and MA1*[3]*. The relative orientation of the heads in* $_{Sm}$*IHM is close to MS03 and 5TBY that were obtained from the IHM model fitted in the map of tarantula striated muscle thick filament. In panels* ***(a)***, ***(b)*** *and* ***(c)****, the structures are superimposed on the motor domain of BH of cardiac models (residues 3-781). The superimpositions are shown in* ***Supplementary Movie 4****.* ***(d)*** *Shows the sequence alignment of human (Homo sapiens) cardiac myosin ELC (MYL3); chicken (Gallus gallus) Smooth muscle myosin ELC (MYL6) and tarantula (genus Aphonopelma) striated muscle ELC (primary accession A0A140UGH4_9ARAC). Tarantula and smooth muscle ELC have no ELC N-terminus extension (*$_{ELC}$*Nterm).*

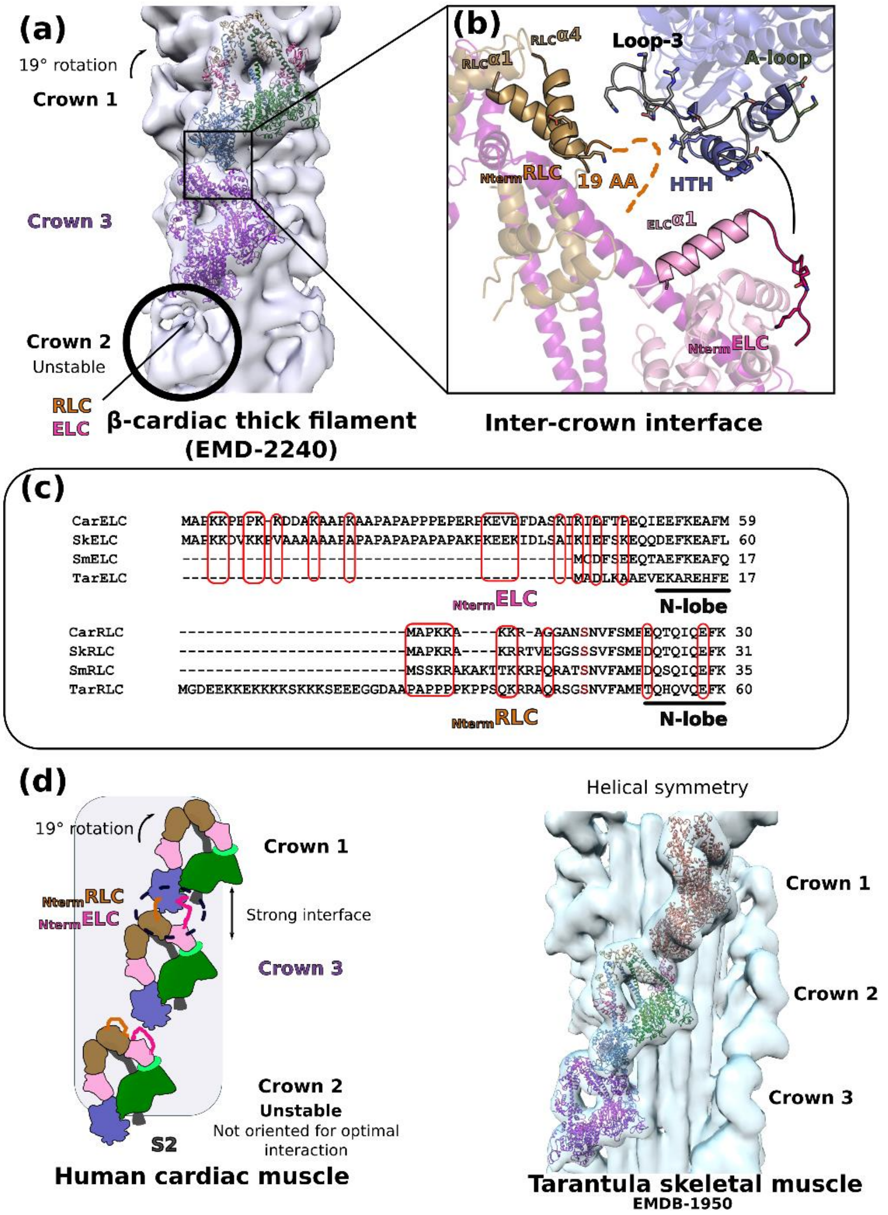

<u>Supplementary Figure 11 – Organization of the thick filament.</u> ***(a)*** *Fit of the IHM motifs in crowns 1 and 3 using the 28 Å resolution map of the relaxed human cardiac thick filament (EMD-2240* [8]*). Crown 2 is less stabilized and less defined in density. A tilt of 19° is observed for crown 1 compared to crowns 2 and 3.* ***(b)*** *Zoom in the interface between crown 1 and 3. Our model indicates that the BH-ELC N-lobe and the N-terminal extensions (Nterm extensions) of both the BH-ELC and the BH-RLC of crown 3 could interact with the L50 helix-turn-helix (HTH) and loop 3 of crown 1. The 49 amino-acid Nterm extension of the ELC can reach the activation loop (A-loop) and the HTH. Since the BH-RLC N-term is not involved in interactions at the interface between the two RLCs, the phosphorylation site could modulate the IHM stability mostly via these inter-crown interactions in cardiac muscle, unlike what occurs for smooth muscle myosin.* ***(c)*** *Analysis of the sequence conservation of the $_{Nterm}$ELC and $_{Nterm}$RLC in human cardiac myosin (Car), human skeletal myosin (Sk), smooth muscle myosin (Sm) and Aphonopelma tarantula myosin (Tar). Charged regions are in a red box, the position of the phosphorylatable serine is colored in red.* ***(d)*** *Schematic overview of the differences stabilizing the sequestered states in human cardiac and tarantula striated muscle. In cardiac muscle (left), the inter-crown interface stabilizes the IHM of crowns 1 and 3, while crown 2 is less stabilized and less defined in the density because (i) it is not oriented to establish this interface and (ii) the crown 2/crown 3 inter-crown distance is longer compared to crown 3/crown 1. A fit of the IHM cardiac model in the purely helical relaxed tarantula striated muscle*[9,10,11] *(right). Compared to cardiac myosin, tarantula myosin has a longer $_{Nterm}$RLC and a shorter $_{Nterm}$ELC. Differences in both sequence and symmetry imply that the inter-crown interface greatly differ in cardiac and tarantula fibers, even though they can involve the same structural elements.*

Supplementary Tables

Supplementary Table 1 – EM validation

IHM β-cardiac myosin (EMD-15353)	
**Data collection and processing**	
Microscope	Titan KRIOS G3
Detector	K3
Magnification	105k
Total exposure (e-/Å^2)	39.2
Exposure time	2.4 s
Electron exposure per frame (e-/Å^2)	0.98
Defocus range (μm)	-0.8, -2.2
Pixel size (Å)	0.84
Symmetry imposed	C1
Initial particles images (no.)	493179
Final particle images (no.)	213596
Map resolution (Å)	3.6
FSC threshold	0.143
Map resolution range (overall)	3-9
**Refinement**	
Initial model used (PDB codes)	5N69, 2XFM, homology modeling from 7MF3
Map sharpening B-factor (Å^2)	67.4
Model composition	
Non-H atoms	18839
Protein residues	2322
Ligands	6
RMSD from ideal geometry	
Bond length (Å)	0.002
Bond angles (°)	0.601
Validation	
Molprobity score	1.87
clashcore	16.05
Ramachandran (%)	
Outliers	0
Allowed	2.89
Favored	97.11
Validation	
CC (mask)	0.79
CC (volume)	0.78
EMRinger score (overall mode)	0.81
EMRinger score (1-786, heads regions at high resolution)	1.15

IHM β-cardiac myosin head-head masked map (EMD-15354)	
**Data collection and processing**	
Microscope	Titan KRIOS G3
Detector	K3
Magnification	105k
Total exposure (e-/Å^2)	39.2
Exposure time	2.4 s
Electron exposure per frame (e-/Å^2)	0.98
Defocus range (µm)	-0.8, -2.2
Pixel size (Å)	0.84
Symmetry imposed	C1
Initial particles images (no.)	493179
Final particle images (no.)	213596
Map resolution (Å)	3.2
FSC threshold	0.143
Map resolution range (overall)	2.5-6
**Refinement**	
CC (mask)	0.61
CC (volume)	0.61
EMRinger score (1-786, heads regions at high resolution)	1.78

Supplementary Table 2 – Interactions stabilizing cardiac IHM

This table details the residues involved in the different interfaces of the cardiac IHM in the columns on the left and compares with the residues found in similar contacts for stabilization of the smooth IHM on the left. In particular, an orange background allows to find which interactions are drastically different between the smooth and the cardiac IHMs. The five interfaces are listed one after the other, with subdivisions for the Head/Head interface so that the reader can easily find which residues correspond to the different parts of the myosin head involved in these interactions.

Interactions that differ drastically between smooth and cardiac are shown with orange background.

(i) Head-Head interactions

BH HCM-loop / FH Transducer (HO-linker and β-bulge)<br>The BH HCM loop is positioned differently on the FH head in Cardiac and Smooth IHMs. It interacts mostly with the Transducer in Cardiac while in Smooth, interactions reach the HD-linker but no interaction occur with the N-terminus part of the HO-linker			
**BH**	**FH**	**SmHMM**	**HCM/effect**
HCM-loopR403	HO-linkerQ454 (Mesa) (electrostatic)<br>HO-linkerY455 (Mesa) (stacking + electrostatic)<br>B-bulgeA254 (Mesa)	BH-HCMR406, K408<br>FH-HO-linkerQ456-G457 (electrostatic)<br>FH-HD-linkerR168,E169	R403Q [12]<br>R403L [13]<br>R403W [14]<br>R403G [15]
HCM-loopN408	HO-linkerK450 (Mesa) (electrostatic)<br>HO-linkerP452 (Mesa)<br>HO-linkerR453 (Mesa)	**No interaction**<br>for residues BH-HCMR411<br>and residues Nter of the FH-HO.linkerT453,R455	N408K [16]<br>R453C/H/S [17,18,19,20,]
HCM-loopE409	β-bulgeH251 (Mesa) (electrostatic)<br>HO-linkerR453 (Mesa) (electrostatic)<br>HO-linkerQ454 (Mesa)	**No interaction**<br>BH-HCMD412<br>FH- β-bulgeN255	H251N [21,22,4,23]<br>R453C/H/S [17,18,19,20]
HCM-loopY410	HO-linkerQ454 (Mesa)<br>HO-linkerR453 (Mesa)	**Different interaction**<br>BH-HCMV413,Q415<br>FH-HD-linkerE169,R168<br>FH-Transβ5F460	
**BH Loop4 / FH Relay**<br>Equivalent interaction in Cardiac and Smooth IHMs between Relay and Loop4			
Loop4K367 carbonyl	RelayK503 (Mesa) (electrostatic)	BH-Loop4E370, Q375<br>FH-RelayR507 (electrostatic)	
**BH PHHIS / FH Converter**<br>Lock-and-key interactions between the FHConverter T-loop and the BHU50 subdomain involve similar residues but in fact differ as the top-loop conformation differ in the two IHMs. The difference in size of the lock and key residue side chain, the top loop [3] size and composition lead to drastically different interactions between the Converter and the BH head.			
HM-helixD382<br>HM-helixK383	T-loopI736	FH-T-loopF746<br>BH-HM-helix Q385<br>BH-HM-helix K386	I736T[24]<br>D382Y [23]<br>K383N[25]<br>Y386H [26]

HM-helixY386<br>HJ-helixL302		BH-HM-helix H389<br>BH- HJ-helixL306	
**BH PHHIS / FH Converter**<br>**Different positioning of the Converter leads to distinct interactions in Cardiac and Smooth IHMs between the U50 subdomain and the Converter. Several polar interactions are found in Cardiac on either side of the I736 lock and key residue while no polar interaction is predicted at this interface in the smooth IHM on either side of the F746 lock and key residue.**			
HM-helixE379 (electrostatic)<br>HM-helixK383 (electrostatic)<br>HM-helixD382 (electrostatic)<br>HM-helixY386 (electrostatic)	T-loopD737<br>T-loopI736 carbonyl<br>T-loopS738<br>T-loopG733 carbonyl	**No polar interaction** involving the Top loop of Smooth FH head<br>No interaction with G749<br>No polar interaction	E379K[27]<br>K383N[25]<br>D382Y [23]<br>Y386H [26]<br>G733E [4]<br>Q734E/P[28,29]
HM-helix T378.E379.D382<br>HM-helixY386	T-loopD737.S738 apolar interactions<br>T-loopQ734	BH-HM-helix N381.T382.Q385<br>FH-Top-loopM747$_{mc}$D748$_{mc}$	
HN-helixN391 (long range)<br>HN-helixA393<br>No interaction via K397 and converter<br>HM-helixT378	ConvQ720<br>ConvD717<br>ConvG716<br>ConvL714<br>ConvT761<br>ConvH760	BH- HN-helix T396<br>BH- HN-helix R400 (electrostatic) ONLY IN SMOOTH<br>FH-ConvE729 (electrostatic)<br>FH-ConvV726<br>FH-ConvQ728 (electrostatic)<br>FH-ConvK773 (electrostatic, long range)	G716A[27]
**BH PHHIS / FH HD-linker and ELC**<br>**The FH HD-linker interacts with the U50 subdomain but make only non polar interactions in Smooth IHM as it is more involved in interactions with the HCM-loop**			
HN-helix D394.K397	HD-linkerR169 (Mesa) (electrostatic)	BH-HN-helixD397 (long range)<br>BH- HN-helix R400<br>FH-HD-linkerR168<br>Not defined in density but consistent	D394E[27]<br>R169G
HU-helix K611	ELC-loop3Res143-145	**No interaction**	R143G/Q [29]

(ii) BH - S2 Coiled-coil interactions

**No Conservation between Smooth and Cardiac : Seg3 of Smooth interacts with only a part of the HO-linker of the BH head to mimic some of the interactions that involve the proximal S2 region of Cardiac**			
**BH Mesa**	**Coiled-coil**	**SmHMM**	**HCM**
$_{\text{HO-linker}}$K450 (Mesa)<br>$_{\text{HO-linker}}$Q451 (Mesa)	$_{\text{S2}}$E874 (electrostatic)<br>$_{\text{S2}}$V878	Interaction with Seg3	
$_{\text{HO-linker}}$P452.R453.Q454 (Mesa)	$_{\text{S2}}$L881-Q882<br>$_{\text{S2}}$M877<br>$_{\text{S2}}$V878	Interaction with Seg3	Q882E [30]<br>R453C/H/S [17,18,19,20]
$_{\text{HW-helix}}$K657 (Mesa)	$_{\text{S2}}$L889 (electrostatic, backbone)<br>$_{\text{S2}}$Q892<br>$_{\text{S2}}$A893<br>$_{\text{S2}}$D896	**No interaction**	
$_{\text{HW-helix}}$T660 (Mesa) (electrostatic)<br>$_{\text{HW-helix}}$N661 (Mesa) (electrostatic)<br>$_{\text{HW-helix}}$S664 (Mesa) (electrostatic)	$_{\text{S2}}$Q892	**No interaction**	T660N [16]
$_{\text{HW-helix}}$N656 (Mesa) (electrostatic, backbone)<br>$_{\text{HW-helix}}$T660 (Mesa)	$_{\text{S2}}$D896	**No interaction**	T660N[16]
**No interactions occur in Cardiac IHM between the FH head and the proximal S2 region, whereas a few interactions occur in Smooth IHM**			
**No interaction**		$_{\text{FH-HCM loop}}$R411<br>$_{\text{FH-S2}}$E941.Q945	
		$_{\text{FH-Loop2}}$M655.K652.F656<br>$_{\text{BH-S2}}$E938 (electrostatic)<br>E939.E942	

(iii) RLC/RLC interactions (not conserved between Smooth and Cardiac)

**BH-RLC/BH**	**FH-RLC**	**SmHMM**	**HCM ?**
**The interactions between RLC lobes of the BH and FH heads depend on the presence of the N-terminal extension of the FH head in Smooth, but not Cardiac as the RLCs of the two heads interact directly in Cardiac**			
$_{\text{RLC}\alpha2}$L56, R58<br>$_{\text{IQ}}$ P838<br>$_{\text{RLC}\alpha1}$I35, Q38<br>$_{\text{RLC}\alpha2}$D51, A55<br>$_{\text{IQ}}$ L839	$_{\text{Nterm-ext}}$ M20-F21<br>$_{\text{RLC loop2}}$ N78 (electrostatic) T80<br>$_{\text{RLC}\alpha4}$T80-L83<br>$_{\text{RLC}\alpha1}$K30	$_{\text{FH-Nterm-ext}}$Res15-24<br>Sandwiched between<br>$_{\text{FH-RLC}}$α1- α4<br>and<br>$_{\text{BH-RLC}}$α1- α2 $_{\text{BH-IQ}}$Q852	
$_{\text{IQ}}$R845 (electrostatic)	$_{\text{RLC}\alpha1}$Q27-E28, E31 (electrostatic)	$_{\text{BH-IQ}}$R855 (electrostatic)<br>$_{\text{FH-RLC}\alpha1}$Q27-S28-Q31 (electrostatic)	

(iv) ELC/RLC hinge in the BH and FH heads (not conserved between Smooth and Cardiac)

**BH-ELC**	**BH-RLC**	**SmHMM**	**HCM ?**
$_{\text{ELC- loop1}}$P65-K66	$_{\text{RLC}\alpha 6}$T125-Q126	In the BH lever arm, hydrophobic interactions between:<br>$_{\text{ELC}}$Res L17-R20<br>$_{\text{RLC}}$ResT128-M129-R132<br>$_{\text{IQ}}$L819<br><br>$_{\text{ELC}}$D19,D23 (electrostatic)<br>$_{\text{RLC}}$R132<br><br>$_{\text{ELC}}$R20 (electrostatic)<br>$_{\text{RLC}}$E124- T128<br><br>$_{\text{BH-RLC}}$R44/ $_{\text{FH-RLC}}$D45 carbonyl (electrostatic)	
$_{\text{ELC- loop1}}$P65 carbonyl (electrostatic)	$_{\text{RLC- linker3}}$R129 (electrostatic)		
$_{\text{ELC-}\alpha 1\text{-}}$ D62 carbonyl (electrostatic)	$_{\text{RLC}\alpha 6}$Q126 (electrostatic)<br>$_{\text{IQ}}$R808 (electrostatic)		ELC A57G, would weaken helix α1[31,32]

**FH-ELC**	**FH-RLC/FH-IQ2**	**SmHMM**	**HCM ?**
$_{\text{ELC- loop1}}$R63 carbonyl<br>$_{\text{ELC- loop1}}$R63	$_{\text{RLC}\alpha 6}$T125 (electrostatic)<br>$_{\text{RLC}\alpha 6}$Q126 (electrostatic)	No clear interaction in the FH lever arm.	
$_{\text{ELC- loop1}}$P65 carbonyl (electrostatic)<br>$_{\text{ELC- loop1}}$K66	$_{\text{RLC- linker3}}$R129		
$_{\text{ELC-}\alpha 1\text{-}}$D62 carbonyl (electrostatic)<br><br>$_{\text{ELC-}\alpha 1\text{-}}$E56 (electrostatic)<br><br>$_{\text{ELC-}\alpha 1\text{-}}$L60	$_{\text{RLC}\alpha 6}$T125 carbonyl (electrostatic)<br><br>$_{\text{IQ}}$R808 (electrostatic)<br>$_{\text{IQ}}$L805<br><br>$_{\text{IQ}}$L804		ELC A57G, would weaken helix α1 [31,32]

Supplementary References

1. Heissler, S. M., Arora, A. S., Billington, N., Sellers, J. R. & Chinthalapudi, K. Cryo-EM structure of the autoinhibited state of myosin-2. *Sci. Adv.* **7**, eabk3273 (2021).

2. Planelles-Herrero, V. J., Hartman, J. J., Robert-Paganin, J., Malik, F. I. & Houdusse, A. Mechanistic and structural basis for activation of cardiac myosin force production by omecamtiv mecarbil. *Nat. Commun.* **8**, 190 (2017).

3. Robert-Paganin, J., Auguin, D. & Houdusse, A. Hypertrophic cardiomyopathy disease results from disparate impairments of cardiac myosin function and auto-inhibition. *Nat. Commun.* **9**, 4019 (2018).

4. Nag, S. *et al.* The myosin mesa and the basis of hypercontractility caused by hypertrophic cardiomyopathy mutations. *Nat. Struct. Mol. Biol.* **24**, 525–533 (2017).

5. Spudich, J. A. Three perspectives on the molecular basis of hypercontractility caused by hypertrophic cardiomyopathy mutations. *Pflugers Arch.* **471**, 701–717 (2019).

6. Llinas, P. *et al.* How Actin Initiates the Motor Activity of Myosin. *Dev. Cell* **33**, 401–412 (2015).

7. Alamo, L. *et al.* Effects of myosin variants on interacting-heads motif explain distinct hypertrophic and dilated cardiomyopathy phenotypes. *Elife* **6**, 2386–2390 (2017).

8. Al-Khayat, H. A., Kensler, R. W., Squire, J. M., Marston, S. B. & Morris, E. P. Atomic model of the human cardiac muscle myosin filament. *Proc. Natl. Acad. Sci. U. S. A.* **110**, 318–23 (2013).

9. Brito, R. *et al.* A molecular model of phosphorylation-based activation and potentiation of tarantula muscle thick filaments. *J. Mol. Biol.* **414**, 44–61 (2011).

10. Alamo, L. *et al.* Three-Dimensional Reconstruction of Tarantula Myosin Filaments Suggests How Phosphorylation May Regulate Myosin Activity. *J. Mol. Biol.* **384**, 780–797 (2008).

11. Alamo, L. *et al.* Conserved Intramolecular Interactions Maintain Myosin Interacting-Heads Motifs Explaining Tarantula Muscle Super-Relaxed State Structural Basis. *J. Mol. Biol.* **428**, 1142–1164 (2016).

12. Sarkar, S. S. *et al.* The hypertrophic cardiomyopathy mutations R403Q and R663H increase the number of myosin heads available to interact with actin. *Sci. Adv.* **6**, eaax0069 (2020).

13. Charron, P. *et al.* Prenatal molecular diagnosis in hypertrophic cardiomyopathy: report of the first case. *Prenat. Diagn.* **24**, 701–703 (2004).

14. Keller, D. I. *et al.* Human homozygous R403W mutant cardiac myosin presents disproportionate enhancement of mechanical and enzymatic properties. *J. Mol. Cell. Cardiol.* **36**, 355–362 (2004).

15. Alamo, L., Pinto, A., Sulbarán, G., Mavárez, J. & Padrón, R. Lessons from a tarantula: new insights into myosin interacting-heads motif evolution and its implications on disease. *Biophys. Rev.* **10**, 1465–1477 (2017).

16. Bos, J. M. *et al.* Characterization of a Phenotype-Based Genetic Test Prediction Score for Unrelated Patients With Hypertrophic Cardiomyopathy. *Mayo Clin. Proc.* **89**, 727–737 (2014).

17. Bloemink, M. *et al.* The Hypertrophic Cardiomyopathy Myosin Mutation R453C Alters ATP Binding and Hydrolysis of Human Cardiac β-Myosin. *J. Biol. Chem.* **289**, 5158–5167 (2014).

18. Sommese, R. F. *et al.* Molecular consequences of the R453C hypertrophic cardiomyopathy mutation on human -cardiac myosin motor function. *Proc. Natl. Acad. Sci.* **110**, 12607–12612

(2013).

19. Spudich, J. A. *et al.* Effects of hypertrophic and dilated cardiomyopathy mutations on power output by human β-cardiac myosin. *J. Exp. Biol.* **219**, 161–7 (2016).

20. Yu, C.-M. *et al.* Left ventricular reverse remodeling but not clinical improvement predicts long-term survival after cardiac resynchronization therapy. *Circulation* **112**, 1580–1586 (2005).

21. Pablo, K. J. *et al.* Prevalence of Sarcomere Protein Gene Mutations in Preadolescent Children With Hypertrophic Cardiomyopathy. *Circ. Cardiovasc. Genet.* **2**, 436–441 (2009).

22. Adhikari, A. S. *et al.* Early-Onset Hypertrophic Cardiomyopathy Mutations Significantly Increase the Velocity, Force, and Actin-Activated ATPase Activity of Human β-Cardiac Myosin. *Cell Rep.* **17**, 2857–2864 (2016).

23. Adhikari, A. S. *et al.* β-Cardiac myosin hypertrophic cardiomyopathy mutations release sequestered heads and increase enzymatic activity. *Nat. Commun.* **10**, 2685 (2019).

24. Kawana, M., Sarkar, S. S., Sutton, S., Ruppel, K. M. & Spudich, J. A. Biophysical properties of human beta-cardiac myosin with converter mutations that cause hypertrophic cardiomyopathy. *Sci Adv* **3**, e1601959 (2017).

25. Kuang, S. Q. *et al.* Identification of a novel missense mutation in the cardiac beta-myosin heavy chain gene in a Chinese patient with sporadic hypertrophic cardiomyopathy. *J. Mol. Cell. Cardiol.* **28**, 1879–1883 (1996).

26. Millat, G. *et al.* Prevalence and spectrum of mutations in a cohort of 192 unrelated patients with hypertrophic cardiomyopathy. *Eur. J. Med. Genet.* **53**, 261–267 (2010).

27. Kassem, H. S. *et al.* Early Results of Sarcomeric Gene Screening from the Egyptian National BA-HCM Program. *J. Cardiovasc. Transl. Res.* **6**, 65–80 (2013).

28. Nanni, L. *et al.* Hypertrophic cardiomyopathy: two homozygous cases with 'typical' hypertrophic cardiomyopathy and three new mutations in cases with progression to dilated cardiomyopathy. *Biochem. Biophys. Res. Commun.* **309**, 391–8 (2003).

29. Song, L. *et al.* Mutations profile in Chinese patients with hypertrophic cardiomyopathy. *Clin. Chim. Acta* **351**, 209–216 (2005).

30. Mohiddin, S. A. *et al.* Utility of genetic screening in hypertrophic cardiomyopathy: prevalence and significance of novel and double (homozygous and heterozygous) beta-myosin mutations. *Genet. Test.* **7**, 21–27 (2003).

31. Huang, W. & Szczesna-Cordary, D. Molecular mechanisms of cardiomyopathy phenotypes associated with myosin light chain mutations. *J. Muscle Res. Cell Motil.* **36**, 433–445 (2015).

32. Sitbon, Y. H. *et al.* Cardiomyopathic mutations in essential light chain reveal mechanisms regulating the super relaxed state of myosin. *J. Gen. Physiol.* **153**, (2021).
