# Supplementary material for: Cryo-EM structure of the folded-back state of human β-cardiac myosin
Source: Nat Commun. 2023 May 31;14:3166. doi: 10.1038/s41467-023-38698-w (PMC10232470; doi:10.1038/s41467-023-38698-w)
Supplement: Supplementary file 3 — Description of additional supplementary files [file 41467_2023_38698_MOESM3_ESM.pdf]

Description of additional supplementary files

Supplementary Movie 1: overview on the map 1 (EMD-15353) at a global resolution of 3.6 Å. The blocked head is colored in green, the free head is colored in blue. The ELCs are colored in pink and the RLCs are colored in light brown. Different views are represented, the final view displays the head-head interface with FHTop-Loop (orange), BHLoop-4 (cyan) and BHHCM-loop (red).

Supplementary Movie 2: overview on the map 2 (EMD-15354) at a global resolution of 3.2 Å. The blocked head is colored in green, the free head is colored in blue. The ELCs are colored in pink and the RLCs are colored in light brown. Different views are represented. In the views of the active site, Switch-1 is colored in black, Switch-2 is colored in orange, P-loop is colored in purple. The final view displays the head-head interface with FHTop-Loop (orange), BHLoop-4 (cyan) and BHHCM-loop (red).

Supplementary Movie 3: Illustration of the asymmetry of the RLC-RLC interface. See Supplementary Fig. 8.

Supplementary Movie 4: Comparison of the cardiac and smooth muscle IHM. Illustration of the differences in the positioning of the two heads of the IHM. See Fig. 3a. Cardiac muscle IHM (BH:green, FH:blue) is compared to Smooth muscle IHM (yellow) after super-imposition on the BH. The movie then shows how the Cardiac muscle low resolution model (5tby, red) compares to the Cardiac structure. Note that when the BH are aligned, the FH of the cardiac model differ greatly from the FH of the cardiac cryo-EM structure, but is wrongly positioned in fact where the FH of the Smooth muscle IHM is found.
