# Supplementary material for: Cryo-EM structure of the folded-back state of human β-cardiac myosin
Source: Nat Commun. 2023 May 31;14:3166. doi: 10.1038/s41467-023-38698-w (PMC10232470; doi:10.1038/s41467-023-38698-w)
Supplement: Supplementary file 8 — Reporting Summary [file 41467_2023_38698_MOESM8_ESM.pdf]

nature portfolio

Corresponding author(s): Julien Robert-Paganin, Kathleen Ruppel, Anne Houdusse

Last updated by author(s): May 11, 2023

Reporting Summary

Nature Portfolio wishes to improve the reproducibility of the work that we publish. This form provides structure for consistency and transparency in reporting. For further information on Nature Portfolio policies, see our Editorial Policies and the Editorial Policy Checklist.

Statistics

For all statistical analyses, confirm that the following items are present in the figure legend, table legend, main text, or Methods section.

n/a	Confirmed	
x	☐	The exact sample size (*n*) for each experimental group/condition, given as a discrete number and unit of measurement
x	☐	A statement on whether measurements were taken from distinct samples or whether the same sample was measured repeatedly
x	☐	The statistical test(s) used AND whether they are one- or two-sided<br>*Only common tests should be described solely by name; describe more complex techniques in the Methods section.*
x	☐	A description of all covariates tested
x	☐	A description of any assumptions or corrections, such as tests of normality and adjustment for multiple comparisons
x	☐	A full description of the statistical parameters including central tendency (e.g. means) or other basic estimates (e.g. regression coefficient) AND variation (e.g. standard deviation) or associated estimates of uncertainty (e.g. confidence intervals)
x	☐	For null hypothesis testing, the test statistic (e.g. *F*, *t*, *r*) with confidence intervals, effect sizes, degrees of freedom and *P* value noted<br>*Give P values as exact values whenever suitable.*
x	☐	For Bayesian analysis, information on the choice of priors and Markov chain Monte Carlo settings
x	☐	For hierarchical and complex designs, identification of the appropriate level for tests and full reporting of outcomes
x	☐	Estimates of effect sizes (e.g. Cohen's *d*, Pearson's *r*), indicating how they were calculated

Our web collection on statistics for biologists contains articles on many of the points above.

Software and code

Policy information about availability of computer code

Data collection	No software used.
Data analysis	Motion correction: MotionCorr2 (version 1.6.4). CryoEM data processing: Gctf (version 1.18), cryoSPARC (version 4.0).<br>Model building and refinement: Phenix (version 1.19.2-4158-0000), Coot (version 0.9.2), Swiss-MODEL online (https://swissmodel.expasy.org/interactive).<br>Molecular dynamics: GROMACS (version 2018.3), CHARM-GUI (version 3.7, December 2021).<br>Model validation: Molprobity and EMRinger included in Phenix package (version 1.19.2-4158-0000).

For manuscripts utilizing custom algorithms or software that are central to the research but not yet described in published literature, software must be made available to editors and reviewers. We strongly encourage code deposition in a community repository (e.g. GitHub). See the Nature Portfolio guidelines for submitting code & software for further information.

Data

Policy information about availability of data

All manuscripts must include a data availability statement. This statement should provide the following information, where applicable:

- Accession codes, unique identifiers, or web links for publicly available datasets
- A description of any restrictions on data availability
- For clinical datasets or third party data, please ensure that the statement adheres to our policy

The atomic model is available in the PDB 72 under the code 8ACT [PDB DOI: https://doi.org/10.2210/pdb8ACT/pdb]. The cryo-EM maps Map 1 and Map 2 are available in the EMDB database Patwartdhan et al., Methods Enzymol, 2016 under the accession numbers EMD-15353 [https://www.ebi.ac.uk/pdbe/entry/emdb/EMD-15353] and EMD-15354 [https://www.ebi.ac.uk/pdbe/entry/emdb/EMD-15354] respectively. The homology model 5TBY is available in the PDB [https://doi.org/10.2210/pdb5TBY/pdb]. The homology model MS03 is available for download on the website of Jim Spudich's lab (http://spudlab.stanford.edu/homology-models/). The homology model MA1 is available for download as Supplementary Data 2 in Robert-Paganin et al., Nat Comms, 2018.

Human research participants

Policy information about studies involving human research participants and Sex and Gender in Research.

Reporting on sex and gender	No human research in this work.
Population characteristics	Not applicable.
Recruitment	Not applicable.
Ethics oversight	Not applicable.

Note that full information on the approval of the study protocol must also be provided in the manuscript.

Field-specific reporting

Please select the one below that is the best fit for your research. If you are not sure, read the appropriate sections before making your selection.

☒ Life sciences ☐ Behavioural & social sciences ☐ Ecological, evolutionary & environmental sciences

For a reference copy of the document with all sections, see nature.com/documents/nr-reporting-summary-flat.pdf

Life sciences study design

All studies must disclose on these points even when the disclosure is negative.

Sample size	Not applicable.
Data exclusions	Not applicable.
Replication	Not applicable.
Randomization	Not applicable.
Blinding	Not applicable.

Reporting for specific materials, systems and methods

We require information from authors about some types of materials, experimental systems and methods used in many studies. Here, indicate whether each material, system or method listed is relevant to your study. If you are not sure if a list item applies to your research, read the appropriate section before selecting a response.

Materials & experimental systems

n/a	Involved in the study	
☒	☐	Antibodies
☐	☒	Eukaryotic cell lines
☒	☐	Palaeontology and archaeology
☒	☐	Animals and other organisms
☒	☐	Clinical data
☒	☐	Dual use research of concern

Methods

n/a	Involved in the study	
☒	☐	ChIP-seq
☒	☐	Flow cytometry
☒	☐	MRI-based neuroimaging

Eukaryotic cell lines

Policy information about cell lines and Sex and Gender in Research

Cell line source(s)	Differenciated mouse myoblast: C2C12 cells. Purchased from ATCC.
Authentication	Cell line was not authenticated. Commercial cell line.
Mycoplasma contamination	Not tested for mycoplasma contamination.
Commonly misidentified lines (See ICLAC register)	No commonly misidentified cell line was used in the study.
